#  Algorithm for the Construction of a Global Enzymatic Network to be Used for Gene Network Reconstruction

**DOI:** 10.2174/1389202915666140807004909

**Published:** 2014-10

**Authors:** Andrés Quintero, Jorge Ramírez, Luis Guillermo Leal, Liliana López-Kleine

**Affiliations:** 1 Universidad Nacional de Colombia, Department of Biology, Master Student, Universidad Nacional de Colombia-Sede Bogotá; 2 Universidad Nacional de Colombia, School of Mathematics, Associate Professor, Universidad Nacional de Colombia-Sede Medellín; 3 Universidad Nacional de Colombia, Department of Statistics, Master Student, Universidad Nacional de Colombia-Sede Bogotá; 4 Universidad Nacional de Colombia, Department of Statistics, Associate Professor, Universidad Nacional de Colombia-Sede Bogotá, Colombia

**Keywords:** EC number, Gene network reconstruction, Global enzymatic network, KEGG, Perl.

## Abstract

Relationships between genes are best represented using networks constructed from information of different types, with metabolic information being the most valuable and widely used for genetic network reconstruction. Other types of information are usually also available, and it would be desirable to systematically include them in algorithms for network reconstruction. Here, we present an algorithm to construct a global metabolic network that uses all available enzymatic and metabolic information about the organism. We construct a global enzymatic network (GEN) with a total of 4226 nodes (EC numbers) and 42723 edges representing all known metabolic reactions. As an example we use microarray data for *Arabidopsis thaliana* and combine it with the metabolic network constructing a final gene interaction network for this organism with 8212 nodes (genes) and 4606,901 edges. All scripts are available to be used for any organism for which genomic data is available.

## INTRODUCTION

One of the major goals in systems biology is to understand how functional relationships between genes under specific conditions determine changes in the organism's behavior and cell physiology. Co-expression networks and genome-scale metabolic models, have been successfully applied to advance this kind of biological knowledge [1-4].

There are many strategies which allow the construction of such co-expression networks [1-3], however, only a few of those are designed to integrate more than one type of genomic data [4-8], namely: gene expression, sequence homology, cell location, vicinity of chromosomal genes, sites binding to transcription factors, fusion events and phylogenetic profiles. Although all these types of data provide information for the construction of networks, the most valuable type are metabolic networks, since they directly give information on the relationship between cellular entities (enzymes) and metabolites. Moreover, metabolic reactions are global for all organisms and therefore a global metabolic network containing all known metabolic reactions is of special interest for biological network reconstruction of any kind.

One technique that has been implemented for the reconstruction of metabolic networks, and one that integrates various types of genomic data is Kernel Canonical Correlation Analysis (KCCA) [6, 8, 9]. One major difficulty with KCCAoccurs, however, in the necessary tuning of arbitrary parameters (for example thresholds) for the network construction. Methodologies have also been developed for integrating genomic data types into partially known networks [10], generating networks of lesser genomic coverage. Similarly, other proposed methodologies are able to add similarity results on networks established previously as gold standards [9]. Other techniques rely on probabilistic approaches such as Boolean or Bayesian networks [11] or fully probabilistic descriptions [4], which however, have the drawback of being computationally non-practical for databases with a large number of genes. New and effective methodologies that integrate different types of genomic data are therefore needed. 

Of particular interest would be novel approaches that do not depend on arbitrary parameters and that are applicable to a large number of organisms. Such flexible and relatively simple methods will help advance biological knowledge of both model and less studied organisms through the generation of functional gene predictions leading to the formulation of new biological hypotheses.

In this work we propose a general methodology for constructing gene networks using information on metabolic reactions and gene expression data. Our strategy follows the following basic steps: *i)* construction of the global enzymatic network (GEN); *ii)* construction of the organismic enzymatic network (OEN) using a gene similarity matrix based on expression data of an organism of interest; *iii)* integration of both networks in order to obtain a final weighted organismic enzymatic network (WOEN). The implementation of the methodology is available under request as Perl scripts.

To illustrate the proposed methodology, we consider the model organism *Arabidopsis thaliana* and construct WOEN using microarray data and the GEN constructed separately. The used GEN has a total of 4226 nodes (EC numbers) and 42723 edges, and the final WOEN of *Arabidopsis thaliana *has 8212 nodes (genes) and 4606,901 edges, the increase in the number of nodes between the GEN and the WOEN, is explained by the fact that in an organism more than one enzyme can have the same enzymatic activity. The GEN we construct here can be used in combination with gene expression data from the same or any other organism in order to construct the associated gene interaction networks. In that sense, we are using the GEN as a starting point in order to obtain WOENs representing gene coregulations that will depend on the microarray data used. Moreover, any other genomic data type that is also representable as a similarity matrix can be included in the construction by combination with the GEN and could therefore enrich the obtained WOEN.

## MATERIALS AND METHODS

### Genomic Databases

The Genome Expression Omnibus at NCBI (http:// www.ncbi.nlm.nih.gov/geo/) was queried for microarray datasets of the model plant *Arabidopsis thaliana*. A total of sixteen experiments from Arabidopsis in response to several pathogens were used (accession numbers: GSE28800, GSE26973, GSE5513, GSE28800, GSE5752, GSE5513, GSE8319, GSE5752, GSE12856, GSE8319, GSE13739, GSE12856, GSE14961, GSE13739, GSE15236, GSE14961, GSE16472, GSE15236, GSE16497, GSE16472, GSE17382, GSE16497, GSE17875, GSE17382, GSE19273, GSE17875, GSE20188, GSE19273, GSE21920 and GSE20188).

In addition to the gene expression data, information on all known metabolic reactions was obtained from the Kyoto Encyclopedia of Genes and Genomes (KEGG). The KEGG API was used to download the list of up to date EC numbers, enzymes related to each EC number and enzyme sequence. Perl scripts were developed to connect to the REST based KEGG API service, and download all metabolic information required for the global metabolic network; this ensures that any future users of the scripts will use up to date information.

### Preprocessing of Microarray Data and Gene-gene Similarity Matrix

The downloaded datasets were independently pre-processed for noise reduction, quantile normalization and log2 transformation. The RMA (Robust Multiarray Average) method was applied to Affymetrix data using R affy library [12, 13]. Probe IDs were converted to gene IDs. Single probes that matched more than one gene were removed [14]. For those multiple probes that matched a single gene, the maximum expression among the multiple probes was assigned to the gene as suggested in Dozmorov [15]. Genes common to all databases were used for construction of the gene-gene similarity matrix. Microarrays expression data was used to assess the gene expression similarity matrix (GESM). The similarity in GESM for gene pairs was obtained using the mutual information coefficient [16]. 

### Global Enzymatic Network Construction

Using the names, codes and reactions of biochemical activities performed by enzymes, which are defined by the Enzyme Commission (EC) [17], a global enzymatic network (GEN) was constructed, in which the nodes are enzymatic activities (EC numbers), and two activities are connected by an edge if they share at least one metabolite, either as substrate or product [18].

**Fig. (1):**
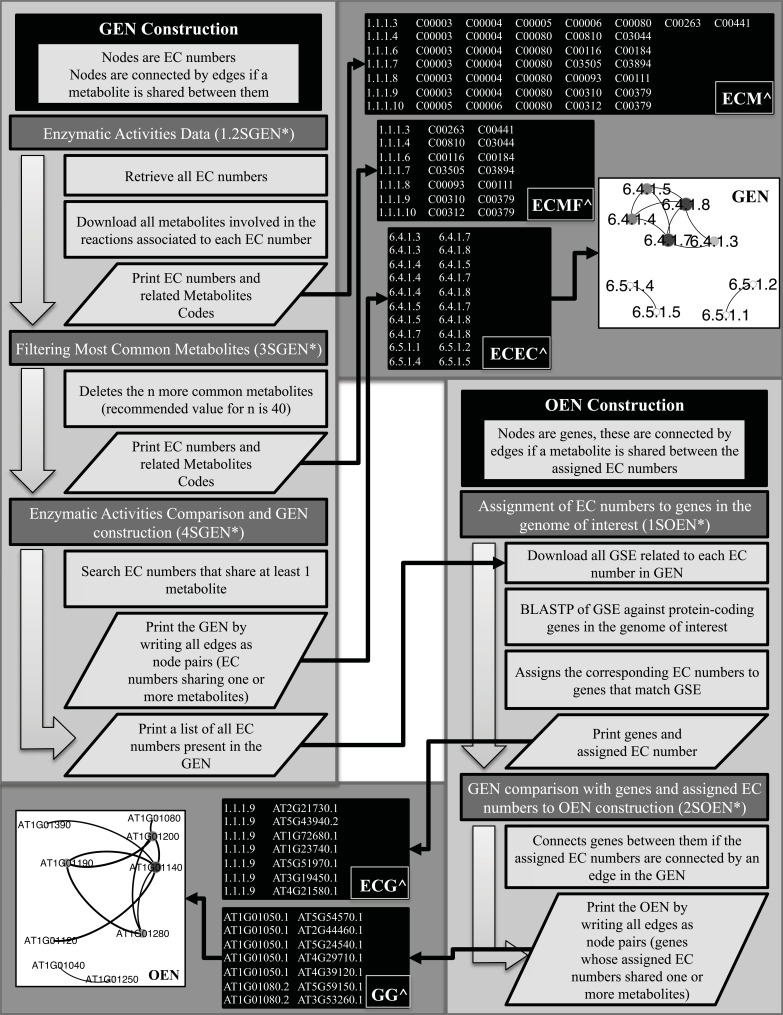
Workflow of the procedure for the GEN and OEN construction. * 1.2SGEN, 3SGEN, 4SGEN, 1SOEN and 2SOEN represent the corresponding abbreviation for the scripts in (Table 1). Black boxes marked with ^ represent examples of the files generated by the related scripts; ECM: first column are EC numbers and the other columns are codified metabolites associated to each EC number; ECMF: ECM after applying the filter for the most common metabolites; ECEC: each line is a pair of EC numbers representing an edge in the GEN; ECG: first column are EC numbers and second column are the genes assigned to each EC number; GG: each line is a pair of genes representing an edge in the OEN.

For the construction of the GEN the following strategy was applied, for which several Perl scripts (Table **1**) were developed (see Fig. **1**, GEN construction):

### 1. Enzymatic Reactions File Construction

A list of all currently known EC numbers was retrieved from the KEGG API, and for each one of the EC numbers, all metabolites involved in the reactions catalyzed by the enzymes annotated with that EC number were downloaded, so that a file with EC numbers and associated metabolites was created. The Perl script used to accomplish this was called “1_Fetch_EC_met.pl” and does not need any input; it connects directly to the KEGG database and retrieves all information needed.

Scripts “1_metabolite_name-code_hash.pl” and “2_reactions_metID.pl” were created for construction of GEN from ftp KEGG data: *i) *“1_metabolite_name-code_hash.pl” uses as input one file with the identification code, name and alternative names of all biological compounds to build a list of equivalences ("dictionary") relating names and alternate names of metabolites with an identification code (used in KEGG). The goal of this step is to create the metabolic network with a less complex and easy to understand nomenclature for future users; *ii) *using the equivalence list, the metabolic reactions were converted into reactions with codified metabolites. Furthermore, as each EC number can have many reaction variants in different organisms, we summarized this information relating each metabolite to one EC number using the main reaction as handle. This step is achieved with the script “2_reactions_metID.pl”, that uses information of each known enzymatic activity (names of the activity, reaction and involved metabolites) and the list containing information on names and codes of metabolites.

### 2. Filtering of Metabolites

As some metabolites, for example H_2_O or NADH, are very common and used in many enzymatic reactions, they have to be withdrawn to avoid an overconnected network. Following Kharchenko [18], the forty most common metabolites were filtered (Supplementary Table **1**). This filtering was done with the perl script “3_filter_reactions _slim.pl”, which allows filtering the first *n *most common metabolites.

### 3. Comparison of Enzymatic Activities and Construction of the GEN

Once the n(=40) most common metabolites were filtered, the GEN was constructed, connecting two nodes (EC numbers) if they share at least one metabolite. This step is achieved using the script “4_network_construction.pl”.

### Organismic Enzymatic Network Construction

In the organismic enzymatic network (OEN) nodes are genes from the genome of the organism of interest, and two genes are connected by an edge if the EC numbers associated to each one of the genes share a metabolite. The following strategy was designed to construct the OEN (Fig. **1**, OEN construction):

### 1. Match of Enzymatic Activities to Genes in the Genome of Interest

The first step, is to assess sequence homology between all known enzymes assigned to each one of the EC numbers (Gold Standard Enzymes (GSE)) [18] present in the GEN and all protein-coding genes in the genome of the organism of interest (Fig. **1**). This step is achieved with the Perl script “1_Fetch_genes.pl”, which downloads from KEGG aminoacid (aa) sequences of all known GSE. Then, this script performs a BLASTP homology search comparing these aa sequences and all known protein-coding genes of the organism of interest. The E-value threshold can be chosen, and for this implementation we used 1x10^-5^_. _Because this search is computationally expensive, the script was developed to run the BLASTP for subsets of EC numbers, providing therefore the option of parallel running over different subsets, and finally combine the results into a single file. The result is a file with a list of protein coding genes and associated EC numbers.

### 2. Construction of the OEN

Once the list of enzymes of the organism of interest has been obtained and related to the corresponding EC numbers of the GEN, the OEN can be constructed (Fig. **1**). The Perl script “2_DataBase_network.pl” does this by searching in the GEN, and linking up genes to define the edges in the OEN, if the associated EC numbers are linked by an edge in the GEN.

### 3. Representation of the OEN as an Adjacency Matrix

Finally, the script “3_Gen-gen_adjacency_matrix.pl” represents the OEN in terms of an adjacency matrix. This matrix is a matrix containing 1 if an edge exists between enzymes coded by the genes of the organism and 0 elsewhere. All protein-coding genes, for which a metabolic activity could not be associated by the BLAST search, are not included in the matrix.

### Weighted Organismic Enzymatic Network Construction

**Fig. (2):**
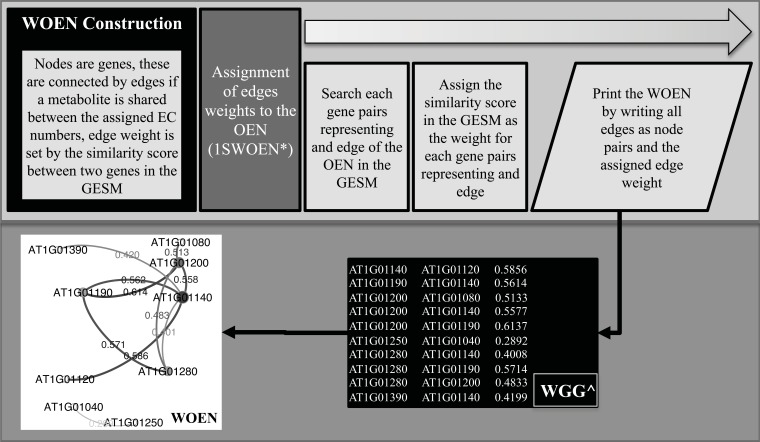
Workflow of the procedure for the WOEN construction. * 1SWOEN represents the corresponding abbreviation for the script in (Table 1). Black box marked with ^ represent an example of the files generated by the related script; WGG: each line is a pair of genes representing an edge with the assigned weight in the WOEN.

At this point a final network (weighted organismic enzymatic network (WOEN)) is obtained combining the OEN and the GESM. This WOEN is represented as an adjacency matrix (Fig. **2**, WOEN construction). Edges could be weighted using the script “4_Weight_adjacency_matrix.pl”.

### Topological Analysis of Networks

Different topological properties of the obtained network were computed in order to reveal changes in the network configuration at each step of our methodology. We consider the networks: GEN, OEN and WOEN; and the following topological properties: number of nodes, number of edges, clustering coefficient, average path length and centralization [19]. This analysis was performed using the R library igraph [20].

### Validation of Edges Linking Immunity Related Genes (IRGs)

The WOEN was mined to validate some functional relationships between immunity related genes (IRGs). Given their importance on immune processes, four IRGs were selected (FLS2, CLV1, RPS2 and RPS4), their edges were filtered and compared with interactions previously reported in literature.

## RESULTS AND DISCUSSION

### GESM for *Arabidopsis thaliana* Microarray Data

After calculating the similarity measurements between all gene pairs, we obtained a GESM of 8,212x8,212 genes for the microarray data of *Arabidopsis thaliana*. This similarity represents the amount of coordinated activity between all pairs of genes and contains all known genes of the organisms, regardless whether their products participate or not in enzymatic activities. Similarities between genes on this matrix strongly depend on the gene expression data used. 

### GEN Construction

**Fig. (3):**
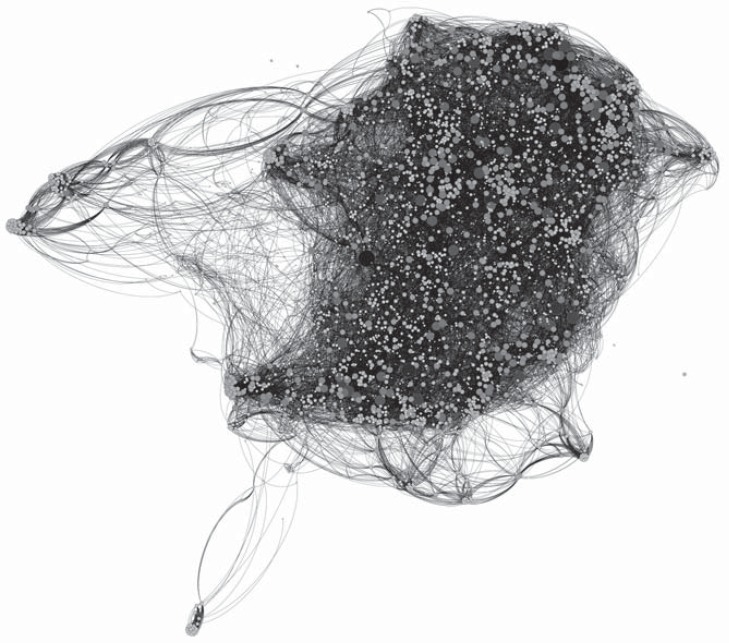
Global metabolic network constructed with the proposed algorithm. Node size and color intensity represent the connectivity of the node. This network has total of 4226 nodes (EC numbers) and 42723 edges and shows a typical scale-free network topology, with a few number of largely connected nodes or hubs (these can be seen as dark grey and black colored nodes), and a large number of nodes with few connections (these can be seen as light grey and white colored nodes). This figure was constructed using Gephi.

Using all enzymatic activities data known to date, a GEN (Table **2**) was constructed (representing 4,226 enzymatic activities, with a final number of 2’043,335 associated GSE in a GEN constructed after filtering for the 40 most common metabolites). The GEN was drawn using Gephi [21] (Fig. **3**). This network is available at, as a list of connected pairs of enzymatic activities.

### OEN and WOEN Construction for *Arabidopsis thaliana*

Using the proposed methodology a OEN and subsequently a WOEN (Table **2**) was constructed for *Arabidopsis thaliana *in order to illustrate the methodology. These networks do not aim to represent overall genetic networks, although if very general gene expression data is used (representing many different conditions), a more general network could be achieved. Nevertheless, a detailed analysis of the network allowed us to retrieve several interesting relationships between genes previously reported in the literature (see section on validation).

### Topological Analysis of Networks

One way of identifying the effect of the different steps of the proposed algorithm is to track the changes in topological properties of the obtained networks (Table **2**). It was found, for example, that GEN is a small graph compared to the subsequent constructed OEN and WOEN. After the BLASTP homology search was performed on GEN, a huge amount of hits per enzyme was revealed. Consequently, the number of edges increased drastically when passing to OEN.

Due to the high number of edges, we expected the OEN to have a higher clustering coefficient; however, during the BLASTP step, the number of nodes augmented simultaneously. As a consequence, both networks exhibit approximately the same degree of clustering. This result supposes that modules of highly connected nodes can be observed indistinctly at the level of enzymes (GEN) or genes (OEN and WOEN).

Same clustering coefficients do not mean equal connectivity in the process represented. To evaluate how edges improve the graph global connectivity, we calculated the centralization and average path length for each network (Table **2**). The higher number of nodes in OEN generated a better connectivity as each pair of nodes is separated by 2 edges contrasting the 4 edges in GEN. The better connectivity found in OEN and WOEN also means that graphs are neither centralized nor hubs-dependent. Besides, in metabolic networks a low average path length is an indicative of more efficient transfer processes [22].

The weighting step reduced the number of nodes and edges in the network. Despite this size reduction, the value of the topological properties of WOEN was about the same. We conclude that genes without expression data are not relevant for the system representation. However, these genes could not be identified using just pathways data. It must be pointed out that expression data allowed us to weight the functional relationships, but also to drop irrelevant nodes from the final WOEN. Finally, our topological analysis results are comparable to those from other methodologies [23].

### Validation of Edges Linking Immunity Related Genes (IRGs) Based on Previous Works

Four of the most important IRGs were searched on the WOEN and their edges were compared with literature (Table **3**). One of them, *FLS2,* was found linked to*BAK1* and *BRI1*. The protein FLS2 is a LRR receptor-like serine/threonine-protein kinase that recognizes peptide from flagellin and triggers plant immunity [24]. On the other hand, BAK1can regulate the tradeoff between immunity and responses to hormones. While BRI1 is a receptor of the growth hormonebrassinosteroid [25]. BAK1 is not only a co-receptor of FLS2 but alto interacts with BAK1 as reported previously [24, 26, 27].

Some *FLS2* edges are also verified by the work of Qi and Tsuda [28]. They propose that FLS2 forms a PTI (MAMP-triggered immunity) signaling complex with RPM1, RPS2 and RPS5 [28]. Lu, Lin, Gao, Wu, Cheng and Avila [29] indicate that PUB12 and PUB13 promote flagellin-induced FLS2 degradation. Besides, the protein PBL1 interacts with FLS2 and they are rapidly phosphorylated upon FLS2 activation by its ligand flg22 [27]. Finally, Mersmann, Bourdais, Rietz and Robatzek [30] validated the ETR1-FLS2 interaction and suggest a requirement of ethylene signaling for FLS2 expression. 

In addition to the RPS2-FLS2 interaction, the complex between RPS2 and ATSK41or AtHIR1 was also reported [31]. RPS2 activates effector-triggered immunity (ETI) after recognizing the bacterial effector protein AvrRpt2. ATSK41 is a hypersensitive protein that is enriched in the plasma membrane. It was identified to be a component of RPS2 complexes [31]. Qi, Tsuda, Nguyen, Wang, Lin, Murphy, Glazebrook, Thordal-Christensen and Katagiri [31] showed that ATSK41 and RPS2 are physically associated and contribute to ETI in presence of *Pseudomonas syringae*pv.tomatoDC3000.

Other edges predicted for *CLV1* and *RPS4* are referred in (Table **3**). CLV1 controls shoot and floral meristem size. Equally, PSY1 is a tyrosine-sulfated peptide hormone. This hormone stimulates cellular proliferation and maintenance of root stem cells [32]. PSY1 and other secreted peptide hormones such as CLE2, suffer post-translational modifications and could function as ligands of CLV1 [32]. Finally, the R protein RPS4 specifies resistance to *Pseudomonas syringae *pv. tomato expressing avrRps4. SGT1 is an ubiquitin ligase-associated protein proved to have a role in host and non-host resistance [33]. The work ofLi, Li, Bi, Cheng, Li and Zhang [34] suggested that SGT1 conforms a complex that negatively regulates RPS4 accumulation. All in all, the functional predictions for these Arabidopsis IRGs are well documented and therefore, the WOENs can be mined for potential IRGs.

## CONCLUSION

The proposed procedure allows obtaining a global enzymatic network and a gene network for any organism for which genomic data is available. Topological analyses showed the graph transformation at each step. The tendency of nodes to cluster remains constant along the process, while an improvement in connectivity and noise reduction was observed after the blast search and expression data integration. The WOEN edges are reinforced with the biological data found in literature. Furthermore, our results from the merging of immunity microarray data and the obtained metabolic network, predict a strong relationship between some genes immune processes in Arabidopsis.

## Figures and Tables

**Table 1.** Description of all the scripts used for the GEN, OEN and WOEN reconstruction. ^scripts written in italics are for users that want to use KEGG ftp database downloaded data; *input and output files names written in quotations marks for easy differentiation with running text, names used can change depending of the user preferences.

Script Abbreviation	Script^	Description	Input Files*	Output Files*
GEN Construction				
1.2SGEN	1-2_Fetch_EC_met.pl	Downloads EC numbers and reactions data and prints a simplified reaction	NA	"reactions_slim"
-	*1_metabolite_name-code_hash.pl*	Creates a equivalence list of complete names and codes for metabolites	Compound information file from KEGG ftp	"metabolite_name_code"
-	*2_reactions_metID.pl*	Prints a simplified reaction	"metabolite_name_code"	"reactions_slim"
3SGEN	3_filter_reactions_slim.pl	Filter n most common metabolites	"reactions_slim"	"reactions_slim.filter_n"
4SGEN	4_network_constuction.pl	Prints the GEN as a list of node pairs	"reactions_slim.filter_n"	"network.reactions_slim.filter_n";"eclist"
OEN Construction				
1SOEN	1_Fetch_genes.pl	Downloads all GSE and BLAST them against genome of interest	"eclist"	"Blasted_genes.list"
2SOEN	2_DataBase_network.pl	Assigns edges within genes comparing associated enzymatic activities in GEN	"Blasted_genes.list";"network.reactions_slim.filter_n"	"Gen-Gen.list"
-	3_Gen-gen_adjacency_matrix.pl	Prints the OEN as an adjacency matrix	"Gen-Gen.list"	"Gen-Gen_adjacency.matrix"
WOEN Construction				
1SWOEN	4_Weight_adjacency_matrix.pl	Weights the OEN using the GESM	"Gen-Gen_adjacency.matrix"; a GESM valid file	"Gen-Gen_similarity.matrix"

**Table 2.** Topological variables measured in the global networks.

Variable	GEN	OEN	WOEN
Nodes	4,226	9,829	8,212
Edges	42,753	6,444,453	4,606,901
Clustering coefficient	0.52	0.48	0.49
Average path length	4.22	2.03	2.02
Centralization	0.05	0.01	0.01

**Table 3.** WOEN edges validated for a selection of IRGs.

IRG	Prediction	References
*FLS2 (AT5G46330)*	*BAK1 (AT4G33430)*	[24, 26]
	*BIK1 (AT2G39660)*	[26, 27]
	*PBL1 (AT3G55450)*	[27]
	*ETR1 (AT1G66340)*	[30]
	*PUB12 (AT2G28830)*	[29]
	*PUB13 (AT3G46510)*	[29]
	*RPM1 (AT3G07040)*	[28]
	*RPS2 (AT4G26090)*	[28]
	*RPS5 (AT1G12220)*	[28]
*CLV1 (AT1G75820)*	*PSY1 (AT1G72300)*	[32]
*RPS2 (AT4G26090)*	*ATSK41 (AT1G09840)*	[31]
	*FLS2 (AT5G46330)*	[28]
*RPS4 (AT5G45250)*	*SGT1A (AT4G23570)*	[34]

## AVAILABILITY AND REQUIREMENTS

Pre-processing of microarray data and similarity measurements between genes based on the gene expression profiles, can be obtained with the aid of several software packages. We recommend using R (R Development Core Team, 2011). Perl is free software and the scripts described in this work can be run in any LINUX/UNIX operating system with a running installation of BLAST+. Scripts are available under request at llopezk@unal.edu.co.

## ABBREVIATIONS

GESM: = Gene Expression Similarity Matrix
GEN: = Global Enzymatic Network
OEN: = Organismic Enzymatic Network
GSE: = Gold Standard Enzymes
aa: = Aminoacid
WOEN: = Weighted Organismic Enzymatic Network
IRGs: = Immunity Related Genes
