# Supplementary material for: Algorithm for the Construction of a Global Enzymatic Network to be Used for Gene Network Reconstruction
Source: Curr Genomics. 2014 Oct;15(5):400–7. doi: 10.2174/1389202915666140807004909 (PMC4245699; doi:10.2174/1389202915666140807004909)
Supplement: Supplementary file 1 [file CG-15-400_SD1.pdf]

Supplementary Material

Algorithm for the Construction of a Global Enzymatic Network to be Used for Gene Network Reconstruction

Andrés Quintero[1], Jorge Ramírez[2], Luis Guillermo Leal[3] and Liliana López-Kleine[4,*]

[1]Universidad Nacional de Colombia, Department of Biology, Master Student, Universidad Nacional de Colombia-Sede Bogotá; [2]Universidad Nacional de Colombia, School of Mathematics, Associate Professor, Universidad Nacional de Colombia-Sede Medellín; [3]Universidad Nacional de Colombia, Department of Statistics, Master Student, Universidad Nacional de Colombia-Sede Bogotá; [4]Universidad Nacional de Colombia, Department of Statistics, Associate Professor, Universidad Nacional de Colombia-Sede Bogotá

Supplementary Table 1. List of the 40 metabolites removed for the GEN construction.

KEGG Entry Code	Metabolite Name	Alternative Name
C00001	H2O	Water
C00002	ATP	Adenosine 5'-triphosphate
C00003	NAD+	NAD
C00004	NADH	DPNH
C00005	NADPH	TPNH
C00006	NADP+	NADP
C00007	Oxygen	O2
C00008	ADP	Adenosine 5'-diphosphate
C00009	Orthophosphate	Phosphate
C00010	CoA	Coenzyme A
C00011	CO2	Carbon dioxide
C00013	Diphosphate	Diphosphoric acid
C00014	Ammonia	NH3
C00015	UDP	Uridine 5'-diphosphate
C00019	S-Adenosyl-L-methionine	S-Adenosylmethionine
C00020	AMP	Adenosine 5'-monophosphate
C00021	S-Adenosyl-L-homocysteine	S-Adenosylhomocysteine
C00022	Pyruvate	Pyruvic acid
C00024	Acetyl-CoA	Acetyl coenzyme A
C00025	L-Glutamate	L-Glutamic acid
C00026	2-Oxoglutarate	Oxoglutaric acid
C00027	Hydrogen peroxide	H2O2
C00028	Acceptor	Hydrogen-acceptor
C00029	UDP-glucose	UDPglucose
C00030	Reduced acceptor	AH2

(Supplementary Table 1) contd….

KEGG Entry Code	Metabolite Name	Alternative Name
C00031	D-Glucose	Grape sugar
C00033	Acetate	Acetic acid
C00035	GDP	Guanosine 5'-diphosphate
C00036	Oxaloacetate	Oxalacetic acid
C00037	Glycine	Aminoacetic acid
C00042	Succinate	Succinic acid
C00043	UDP-N-acetyl-alpha-D-glucosamine	UDP-N-acetyl-D-glucosamine
C00044	GTP	Guanosine 5'-triphosphate
C00055	CMP	Cytidine-5'-monophosphate
C00058	Formate	Methanoic acid
C00060	Carboxylate	R-COOH
C00067	Formaldehyde	Methanal
C00080	H+	Hydron
C00138	Reduced ferredoxin	
C00448	trans,trans-Farnesyl diphosphate	Farnesyl diphosphate
